# Leptin, Leptin Soluble Receptor, and the Free Leptin Index following a Diet and Physical Activity Lifestyle Intervention in Obese Males and Females

**DOI:** 10.1155/2016/8375828

**Published:** 2016-12-06

**Authors:** Jeffrey E. Herrick, Gino S. Panza, Jared M. Gollie

**Affiliations:** Department of Rehabilitation Science, George Mason University, 4400 University Drive, MS 2G7, Fairfax, VA 22030, USA

## Abstract

Leptin (LEP) is associated with appetite regulation and metabolism. Concentration is linear with adiposity, suggesting LEP resistance. LEP circulates freely and bound with its soluble receptor (sOB-r); the ratio is the free leptin index (FLI), an index of leptin resistance; lower FLI suggests reduced biological action.* Purpose*. The aim was to determine the effect of changes in adipose tissue distribution on LEP, sOB-r, and FLI following 6 months (6 M) of a diet/exercise weight loss program (WLP). In addition, we aim to identify predictors of the FLI.* Methods*. 6 M WLP consisted of diet/lifestyle interventions following ADA guidelines. Body composition was assessed by DXA. LEP and sOB-r analysis were done via ELISA.* Results*. 10 adults completed the WLP. Significant reductions were seen in total fat percentage (% fat), nontrunk fat, (NTF), and trunk fat (TF) from base to 3 m and 6 M (*p* ≤ 0.05). The FLI were reduced at 3 M and 6 M for males and 6 M for females. Total body fat and body weight predicted the FLI in both sexes.* Conclusions*. LEP and FLI reductions following 6 M of WLP were achieved independent of sOB-r changes. We also demonstrate that the FLI can be predicted noninvasively through total fat mass and body weight in kilograms.

## 1. Introduction

### 1.1. Leptin and Leptin Soluble Receptor Biology

Leptin (LEP) is an adipokine encoded on the ob gene and is associated with appetite regulation, energy homeostasis [1, 2], immunology [3], and respiration in addition to numerous other biological actions [4]. Leptin concentration is elevated in obesity and positively associated with total body fat [5, 6]. High concentrations of leptin in obesity are indicative of a state of leptin resistance implicating impaired receptor sensitivity and action [7]. Leptin circulates in a free and active form and in a bound and inactive form to its plasma bound soluble receptor (sOB-r) [8]. The (sOB-r) is the cleaved portion of the extracellular domain of a membrane bound leptin receptor and elevated concentrations indicate leptin signaling regulation [6]. The specifics of sOB-r formation in the plasma are not entirely known; however it is theorized that overall elevated leptin concentrations, cellular stress and inflammation, and reduced sOB-r cellular expression may in part mediate serum sOB-r concentrations [9]. In obese adults high levels of serum leptin are believed to reduce sOB-r concentrations and are associated with leptin resistance, although the mechanisms are not entirely understood [10]. Leptin resistance is a robust index of metabolic health and is associated with insulin resistance and may play a role in type II diabetes in addition to substrate regulation [11, 12].

### 1.2. The Free Leptin Index

The free leptin index (FLI) is a biomarker of leptin resistance and the status of leptin action and is the fraction of total leptin and sOB-r concentrations. The FLI is associated with metabolic abnormalities and may be a more potent predictor of developing type II diabetes than traditional biomarkers [11]. Direct measurement of the FLI is costly, time consuming, and not available in every health setting and as a result may limit its usefulness in health monitoring of adults. As such, a noninvasive FLI prediction model that requires easily obtainable measures would be beneficial in providing an additional overall metabolic assessment for adult health, such as while undergoing a weight loss intervention.

### 1.3. Leptin and Gender and Body Fat Distribution

Leptin concentrations are known to be different among males and females [2, 11] which suggests leptin is modulated in some part by unknown sex specific mechanisms, such as body fat patterning or endocrine variations. Nonobese males compared to nonobese females have lower body mass index and lower percent of total body fat in addition to significantly lower leptin and higher sOB-r and FLI [10]. However, in both sexes total body fat mass is positively associated with an elevated free leptin index. Further, it was reported that concentrations of free and bound leptin are greater in obese males and females compared to lean males and females, suggesting both obese sexes have increased proportions compared to their lean peers [13]. Overall, obese males and females share a relative level of elevated leptin resistance despite absolute concentration differences in the plasma of both leptin and its soluble receptor.

### 1.4. Leptin and Leptin Soluble Receptor and Weight Loss

Leptin's response to weight loss through a variety of interventions has been widely investigated [5, 14, 15]. Moderately obese men have reported decreases in plasma leptin following both diet and exercise weight loss programs with significant decreases in the percentage of visceral fat tissue lost [16]. Similarly, in females bound and free leptin are correlated with BMI both before and following weight loss [6]. Also, subcutaneous and visceral adipose tissue changes are associated with reduced leptin; however subcutaneous fat appears to have a larger effect on absolute leptin concentrations [16]. The effect of fat mass loss on sOB-r concentrations is not entirely understood, yet it generally appears as an inverse response to weight and fat mass loss following both weight loss surgery and dietary restriction may exist [17–19]. However, the effects of weight loss and specific body fat distribution changes and sex on sOB-r concentration are not entirely understood.

### 1.5. Statement of Study Purpose

Therefore, the purpose of this study was twofold: first, to determine the effect of changes of total, trunk, and nontrunk fat, on total leptin and sOB-r concentrations, and the free leptin index following 6 months of a lifestyle weight loss intervention in obese males and females; second, to identify a regression model for males and females undergoing a diet and physical activity lifestyle intervention that predicts the free leptin index through commonly available indices of obesity.

## 2. Material and Methods

### 2.1. Subjects

The study group consisted of 7 females and 3 males (55 ± 5 years, 38 ± 16 years, resp.) that participated in a lifestyle weight loss program. All subjects reported to the laboratory in the morning after an overnight fast. All study participants were given an informed consent. The study was approved by the university institutional review board.

### 2.2. Lifestyle Weight Loss Program

The blood and body composition measurements were completed at baseline (0 M), 3 months (3 M), and 6 months (6 M). The weight loss program included reduced caloric intake, educational support, and a general physical activity recommendation. The dietary intervention followed the ADA balanced diet recommendations and was composed of 1250–1500 calories' allotments with a macronutrient profile consisting primarily of 60/20/20 percent of the daily calories from carbohydrates, fats, and proteins, respectively, with ad libitum fruits and veggies consumption (HMR Weight Management Services Corp., Boston, MA). The first 3 M of the active WLP diets were provided for the participants and included a step count goal of 10,000 steps/day. From months 3–6 participants were encouraged to meet the goal of 150–300 minutes of moderate physical activity, which included aerobic and strength training activities. Weekly healthy lifestyle support meetings were provided for participants. The support meetings included diet and nutrition, exercise, and weight loss educational sessions. Participants were required to attend a minimum of 80% of all support meetings.

### 2.3. Body Composition and Anthropometric Analysis

Body composition was assessed by DXA (GE Healthcare Lunar, Buckinghamshire, UK) for total body fat percentage, total fat mass, trunk fat, and nontrunk fat mass. Regions of interest to determine trunk fat were determined by the DXA technician using iDXA software from standard anatomical sites. The anatomical sites included (1) the upper body superior limits of the shoulder, excluding head and neck, (2) lateral boundaries beginning at the glenohumeral joints and continuing along the soft tissue of the trunk with separation between the arms, (3) the inferior boundary of the trunk established from the most superior point of the suprailiac crest. Body composition percentages were generated without utilizing bone mineral content. Nontrunk fat consisted of total body fat in mass minus the trunk fat mass and represented all the fat in the arms, legs, hips, and head.

### 2.4. Leptin and Leptin Soluble Receptor Analysis

A 7 mL fasted blood sample was collected in the morning prior to the start of the intervention (0 M), at the half-way time point (3 M), and at the conclusion of 6 months (6 M) of WLP. The collection was obtained via venipuncture from the antecubital vein into a serum separator tube (Vacutainer, BD Inc., Franklin Lakes, NJ). Following separation the serum samples were parceled into sample tubes and frozen (−80°C) for batch analysis. Leptin (LEP) and soluble receptor (sOB-r) were analyzed via standard Quantikine ELISA methods as per manufacturer specification (R&D Systems, Inc., Minneapolis, MN). Each sample was tested twice and intra-assay correlations of variation were completed. Samples averaged 5.75% with all standard curve correlation coefficients >0.998. The free leptin index (FLI) was determined as the ratio of total leptin and the soluble leptin receptor (LRe) (ng·mL/ng·mL) [20, 21].

### 2.5. Statistical Analysis

Statistical analyses of these data were conducted with SPSS (IBM SPSS Statistical Software V 22, Chicago, IL). Homogeneity of the between-group data was evaluated with Levene's test of equality of variances reporting no significant differences. Independent samples *t*-test was completed for between sex group differences. The within group differences were conducted using analysis of variance (ANOVA) between each time point, 0 M, 3 M, and 6 M. Linear regression modeling was used with all the time points combined (*n* = 30) to determine the primary noninvasive predictors of the FLI which included total fat mass, trunk fat mass, nontrunk fat mass, body fat percentage, and total weight. Leptin and soluble leptin receptor were excluded from the linear regression model testing as they are central to the independent variable we set out to predict. Significance was set at a two-tailed alpha of *p* ≤ 0.05.

## 3. Results

### 3.1. Pre- and Postintervention Changes

Following 6 months of the intervention there were significant reductions in weight, body fat percentage, trunk fat, and nontrunk fat at all time points (Table 1). Total fat was significantly decreased from 0 M to 3 M and 6 M but not from 3 m to 6 M in both males and females. Significant reductions in FLI were found from 0 M to 3 M and 0 M to 6 M in males and 0 M to 6 M only in females (female FLI: 0 M 7.0 ± 2.5, 3 M 4.4 ± 4.0; male FLI: 0 M 6.0 ± 5.9, 3 M 4.0 ± 5.9). There were significant reductions in trunk fat (TF) from 0 M to 3 M in both sexes (female TF 0 M 25.3 ± 5.3, 3 M 20.5 ± 3.7 kg; male TF 0 M 34.8 ± 7.5, 3 M 27.2 ± 12.5 kg).

### 3.2. Free Leptin Index Predictive Modeling

Predictive modeling for the dependent variable FLI was conducted with the independent variables of total weight, truncal fat mass, total fat mass, nontrunk fat mass (total – truncal fat mass in kilograms), and body fat percentage through linear regression. We chose these measures of body composition as they were associated with FLI in our group, are easily obtained in most weight loss settings, and are commonly reported with robust associations to leptin resistance. From our analysis we observed that total body fat was the strongest single predictor for the FLI (*r* = 0.814, *r* ^2^ = 0.662, *p* ≤ 0.001). The model was slightly stronger with the addition of total body weight (*r* = 0.843, *r* ^2^ = 0.711, *p* ≤ 0.001). The addition of weight to the body fat only model was tested with a partial *F*-statistic and the addition was a significant improvement in the estimate of FLI when combined with total body fat mass (partial *F* = 4.59, *p* = 0.04). Truncal fat mass, nontrunk fat mass, and body fat percentage were correlated with FLI but they were not significant contributors to the model testing for predicting the FLI (*r* = 0.719, *r* = 0.779, *r* = 0.597, *p* ≤ 0.05, resp.). Therefore, the regression equation to predict the FLI from total body fat mass in kilograms and body weight in kilograms is below:

(1) FLI=−5.284+0.427 total fat in kg−0.082 body weight in kg.

## 4. Discussion 

We report that a diet and physical activity weight loss intervention produced a significant reduction in the free leptin index in males after 3 months and after 6 months in females. We observed significant reductions in total leptin concentrations after 3 and 6 months of weight loss compared to baseline for both males and females. However there was no change in the soluble leptin receptor concentrations over the course of the weight loss intervention. Our results demonstrated that a change in FLI following a diet and activity intervention was primarily mediated through reduced leptin concentrations and not a change in soluble leptin receptor concentration. Further, we observed that total fat mass was the primary body composition variable that predicted 66% of the FLI and this relationship was slightly more robust at 71% when total body weight was factored in. Our findings suggest that changes in total body fat mass as opposed to changes in any of the measured fat distribution patterns, such as trunk versus nontrunk fat mass, had a significantly more robust effect on the FLI for both males and females. Our findings suggest that truncal fat change is not directly associated with a change in leptin resistance as assessed through the FLI. In addition, we present a noninvasive method to estimate the FLI in males and females undergoing a diet and physical activity weight loss intervention using only the measurements of total body fat and total body weight. Our FLI prediction model provides an additional noninvasive tool in characterizing and tracking individual metabolic health, particularly in obese adults where leptin resistance may indicate risk for numerous health conditions.

Leptin is a fat tissue secreted endocrine factor that is primarily associated with appetite regulation and metabolism in addition to numerous other physiological processes [5]. Leptin concentration in the plasma is positively associated with total fat mass and nearly every measure of obesity in adults [22]. We confirm these findings in our group, as the adults with greatest leptin concentration had the greatest overall total body fat mass, irrespective of sex. Likewise, changes in total body fat were associated with reduced leptin concentration. In contrast, the change in fat mass did not appear to have a significant effect in the concentration of leptin's soluble receptor. In obese adults elevated leptin is an indicator of leptin resistance which is associated with numerous cardiovascular, metabolic, and pulmonary pathomechanisms that negatively affect health [5, 12, 23]. Although we cannot confirm any underlying unreported clinical conditions in our group, we did show that a diet and physical activity lifestyle intervention was sufficient to reduce the FLI, which may suggest an underlying change in leptin action and a positive effect on metabolic mechanisms.

We were surprised that weight loss and changes in body fat did not have a significant effect in altering sOB-r concentrations in either sex over the duration of our 6-month intervention. Indeed, sOB-r concentrations are typically lower in obese adults than lean peers and have been shown to increase following a variety of interventions that reduce overall concentrations in fat mass [17, 19, 24, 25]. Fat mass reductions in obese adults tend to trend toward an equalization in leptin and sOB-r proportions suggesting an improved leptin action following weight loss although absolute concentrations in obese adults undergoing weight loss are not matched to their lean peers [19]. Despite consistent weight and fat mass losses over the duration of the intervention, the male group's leptin concentrations reduced at 3 months with no further reduction observed at 6 months; in contrast the female group's leptin concentration reduced at both 3- and 6-month time points. It is plausible that the male group experienced a rapid decline in leptin at 3 months primarily as a result of the reduced caloric intake and acute anorexia of the dietary proportion of the intervention [10]. As such, it is plausible that the 3-month dietary intervention promoted a state of dietary anorexia that promoted a rapid decline in leptin that may have exceeded the capacity of changes in fat tissue on leptin secretion [10]. In the females where leptin declined significantly throughout the intervention, total fat mass was greater than the male group at all time points, and the proportional changes in fat mass over the duration of the intervention from a larger overall quantity of fat in addition to any acute dietary anorexia may have combined to promote a more persistent decline in circulating leptin over the course of the intervention. However, in either sex, the reduced leptin concentrations appeared to occur independent of any significant change in sOB-r. A reason for this may be the micronutrient proportions of the lifestyle intervention, which were 60% carbohydrate, 20% fat, and 20% protein. It is believed that fat and carbohydrates may act differently on the sOB-r concentration; however the specific actions are not entirely clear in the literature [2]. Indeed, there is no clear consensus on dietary macronutrient content acting on the free leptin index; however it is plausible that dietary fat and carbohydrate proportions may have altered both leptin and sOB-r concentration differently in our groups of obese adults [2, 26–28]. Further study on the impact of dietary macronutrient profile and weight loss on the sOB-r is necessary.

In conclusion, we report that a diet and physical activity lifestyle intervention was sufficient in reducing overall fat mass and subsequently lower circulating leptin and the free leptin index, which may suggest an improvement in leptin action. In contrast, we observed no change in soluble leptin receptor in either sex that suggested that the observed changes in leptin and fat mass distributions were not sufficient to alter soluble receptor concentration as previously reported in the literature. Lastly, we present a prediction model that noninvasively and cheaply predicts the free leptin index from total fat mass and total body weight. Our prediction model may be useful for diet and physical activity intervention programs to employ as a tracking tool for FLI and leptin resistance over the time and provide additional insight into individual metabolic health.

## Figures and Tables

**Table 1:** 

	Male			Female		
	Baseline	3 months	6 months	Baseline	3 months	6 months
Weight (kg)	133.1 ± 24.2	118.3 ± 29.0^*∗*^	113.3 ± 30.9^‡^	102.1 ± 13.0	92.5 ± 10.8^*∗*^	87.3 ± 11.1^*∗*^
Body fat (%)	42.7 ± 2.6	36.4 ± 5.1	33.9 ± 8.1	48.7 ± 2.1	45.4 ± 2.6^*∗*^	42.7 ± 4.0^*∗*^
Total fat (kg)	55.6 ± 14.0	43.4 ± 18.0^*∗*^	40 ± 20.9	48.7 ± 7.5	41.1 ± 6.0^*∗*^	37.4 ± 6.7
Trunk fat (kg)	34.8 ± 7.5	27.2 ± 12.4^*∗*^	22.5 ± 12.6^‡^	25.5 ± 5.5	20.6 ± 3.7^*∗*^	18.2 ± 4.2
Nontrunk fat (kg)	20.8 ± 6.4	16.1 ± 5.6^*∗*^	17.5 ± 8.2	23.2 ± 3.6	20.5 ± 2.5^*∗*^	19.1 ± 2.5^†^
Total lean (kg)	77.5 ± 11.1	75 ± 11.1	73.3 ± 10.3	53.4 ± 6.1	51.4 ± 6.0^*∗*^	49.9 ± 6.5
Trunk lean (kg)	30.2 ± 3.1	31.1 ± 3.6	29.9 ± 3.8	22.9 ± 3.6	22.2 ± 3.3	22.3 ± 3.9
Leptin (ng/mL)	16.9 ± 10.8	10.0 ± 11.8^*∗*^	10.0 ± 10.0^‡^	35.5 ± 13.7	20.9 ± 16.7^*∗*^	16.8 ± 12.2^†^
sOB-r (ng/mL)	3.5 ± 1.0	3.7 ± 1.4	4.1 ± 1.7	5.1 ± 0.9	5.4 ± 1.7	5.5 ± 1.4
FLI	6 ± 5.9	4.1 ± 5.9^*∗*^	3.8 ± 5.0^‡^	7.0 ± 2.5	4.4 ± 4.0	3.7 ± 3.9^‡^

kg = kilogram, nontrunk fat: difference between total fat and trunk fat mass. FLI: free leptin index (LEP/sOB-r).  ^*∗*^Significant difference from baseline to 3 months,  ^†^significant difference from 3 to 6 months, and  ^‡^significant difference from baseline to 6 months. Significance was set to a *p* ≤ 0.05.
